# Acute Pancreatitis, Shock, and Multiple Organ Dysfunction Syndrome in Scrub Typhus

**DOI:** 10.7759/cureus.28233

**Published:** 2022-08-21

**Authors:** Abhin Sapkota, Rahul Devkota, Angel Dongol, Asim Pandey, Tulsiram Bhattarai

**Affiliations:** 1 Internal Medicine, Chirayu National Hospital and Medical Institute Private Limited, Kathmandu, NPL; 2 Department of Medicine, Kathmandu Medical College and Teaching Hospital, Kathmandu, NPL

**Keywords:** medical intensive care unit, shock, scrub typhus, pancreatitis in infections, multiple organ dysfunction

## Abstract

Scrub typhus is an endemic disease caused by the bites of infected chiggers (larval mites) harboring causative bacteria *Orientia tsutsugamushi*. Acute pancreatitis is a rare but occasionally fatal complication of scrub typhus infection caused by vascular and perivascular inflammation of the pancreas.A 34-year-old female presented with severe epigastric pain and multiple episodes of vomiting. Extensive evaluation of the patient revealed acute pancreatitis secondary to scrub typhus. The patient also had septic shock and multiple organ dysfunction syndrome. The patient was mechanically ventilated and treated with doxycycline, and pancreatitis was managed conservatively.

## Introduction

Scrub typhus is a zoonotic infection caused by *Orientia tsutsugamushi*, which is transmitted to the host by bites of infected chiggers (larval mites). It is more prevalent in a large region of Asia known as the “tsutsugamushi triangle,” which includes India, Japan, Indonesia, and other countries. The incubation period for this disease is around 10-12 days. The most common symptoms are high-grade fever, malaise, headache, cough, and generalized lymphadenopathy. At the end of the first week of symptom onset, maculopapular rashes can develop, starting at the trunk and then spreading to the limbs [1]. Systemic complications, such as sepsis, septic shock, multiple organ dysfunction syndrome, hepatitis, acute respiratory distress syndrome, and prerenal azotemia, usually develop after the first week. Abdominal involvement occurs in about 22% of patients in the form of acalculous cholecystitis, gastrointestinal hemorrhage, peritonitis secondary to gastric perforation, and rarely pancreatitis [2,3]. Scrub typhus can have various clinical manifestations and complications that can sometimes involve multiple organ systems, making it very difficult to diagnose and treat.

## Case presentation

A 34-year-old female resident of a rural district in Nepal developed acute unrelenting severe epigastric pain, which was radiating to her back and associated with nausea and a few episodes of vomiting. She also had a fever that was not controlled by oral antipyretics along with yellowish skin discoloration. She had no significant comorbidities. The patient was emergently transferred to the local community hospital in a city near her village. Initially, her vitals were stable, but gradually she became disoriented, tachypneic, and hypotensive with low urine output.

In light of her deteriorating condition, intravenous (IV) fluids, IV piperacillin with tazobactam, and IV ornidazole were started at the community hospital. Her serum amylase was 554 U/L and her serum lipase was 154 U/L, which along with the presence of typical epigastric pain, led to the diagnosis of acute pancreatitis. The serum calcium and lipid profile were normal, which ruled out hyperkalemia and hypertriglyceridemia-induced pancreatitis. She had normal blood sugar and urine ketone was absent. Investigation and prior history ruled out any additional causes of elevated lipase presenting with abdominal pain. She had no past history suggestive of inflammatory bowel disease another possible The significant laboratory investigations were prothrombin time (PT) of 18.0 seconds with an international normalized ratio (INR) of 1.58 and total bilirubin was 2.6 mg/dL with a direct bilirubin of 1.3 mg/dL. Aspartate aminotransferase (AST) was 90 U/L and alanine aminotransferase (ALT) was 190 U/L. Renal function test showed blood urea nitrogen (BUN) of 60 mg/dL and creatinine of 1.7 mg/dL (Table 1). On the third day of admission, she started showing signs of respiratory fatigue and her arterial blood gas analysis showed hypoxemic respiratory failure with a pO2 of 35 mmHg so she had to be intubated. Due to the unavailability of a mechanical ventilator and competent intensive care units (ICU) in her district, she was referred to our hospital. She was brought to Kathmandu in an ambulance while undergoing bag and mask ventilation.

**Table 1:** Laboratory parameters

Investigations	Reference range, adults	At community hospital	At admission to our hospital
Hemoglobin (g/dL)	13.5-17.5	9.1	8.9
White cell count (per μL)	4500-11000	10000	9700
Differential count			
Neutrophils	40-80%	80	78
Lymphocytes	20-40%	19	18
Platelets (per μL)	150000-400000	90000	85000
Amylase (U/L)	40-140	554	473
Lipase (U/L)	8-78	154	130
Liver function tests			
Aspartate aminotransferase (U/L)	5-40	90	68
Alanine aminotransferase (U/L)	5-45	190	105
Total bilirubin (mg/dL)	0.2-1.1	2.6	2
Direct bilirubin (mg/dL)	<0.20	1.3	1.5
Renal function test			
Blood urea (mg/dL)	17-43	60	75
Creatinine (mg/dL)	0.72-1.18	1.7	1.01
C-reactive protein (mg/dL)	<10		203

On initial presentation in the emergency department, she was ill-looking and icteric. Her pulse rate was 120 regular beats per minute, blood pressure was 60/30 mmHg, and respiratory rate was 22 breaths per minute with oxygen saturation maintained at 92% at 5 L/min oxygen given via a nasal cannula. Glasgow Coma Scale (GCS) was 7/15. Abdominal examination revealed diffuse tenderness all over the abdomen, and there was decreased air entry over bilateral lung fields on auscultation.

She was admitted to the surgical ICU. The patient was mechanically ventilated with a fraction of inspired oxygen (FiO2) at 50%. She was started under continuous noradrenaline infusion at the rate of 5 mcg/min. IV fluid support was also given. Due to low hemoglobin of 8.9 gm/dL, a transfusion of two units of whole blood was done. Her C-reactive protein was 203 mg/L, the total count was 9700/μL with neutrophils at 78%, lymphocytes at 18%, and the platelet count was 85000/μL. Her random blood glucose was 30 mg/dL and her repeat serum amylase level was 473 U/L with a serum lipase level of 130 U/L (Table 1). IV ampicillin with sulbactam 1.5 gm thrice daily was started. IV acetaminophen 1 gm once daily and regular insulin on a sliding scale were also started.

Arterial blood gas analysis revealed metabolic acidosis and her acute physiology, age, chronic health evaluation II (APACHE II) score was 24. Six hours after admission and treatment in the ICU, her blood pressure improved to 110/60 mmHg and she became afebrile. A zoonotic panel was performed, as she belonged to an endemic area. Test results for malaria and leptospirosis were negative, whereas the immunoglobulin M (IgM) antibody for scrub typhus was positive. She was then diagnosed with scrub typhus, and IV doxycycline 100 mg twice daily was added to her antibiotic regimen. No growths were seen in urine and blood culture. CT scan of the abdomen and pelvis showed no significant findings.

**Figure 1:**
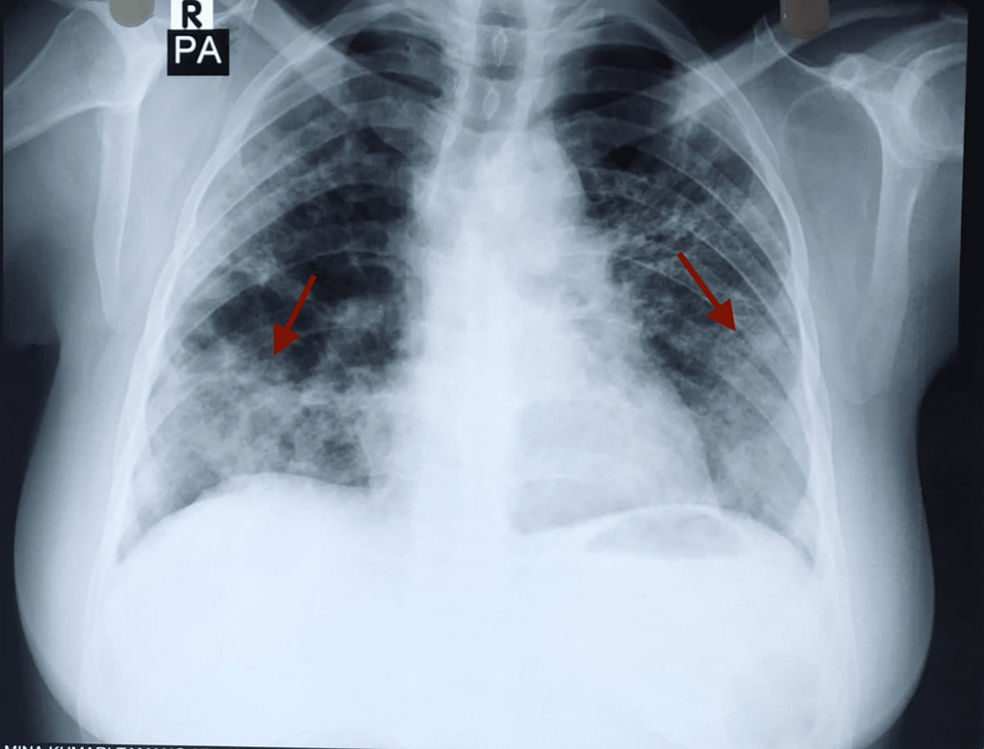
Chest x-ray showing bilateral consolidation

On the sixth day of admission, her body temperature reached 102°F. Her oxygen saturation was 90% under FiO2 of 50%. Her total leucocyte count was 14,300/μL, with neutrophils at 85% and lymphocytes at 10%. Her abdominal pain had resolved and her amylase and lipase levels showed decreasing trend. CSF count was within normal limits and no growths were seen on CSF culture. Chest X-ray showed bilateral consolidation, so she was diagnosed with ventilator-associated pneumonia after ruling out acute respiratory distress syndrome from arterial blood gas analysis (Figure 1). She was managed further with IV meropenem 1 gm thrice daily in addition to IV doxycycline, antipyretics, and fluids. She underwent a tracheostomy on the eighth day of admission under general anesthesia to prevent the complications of prolonged intubation. She was transferred to the medical ICU, a different unit from the surgical ICU where she was initially admitted. After nine days of stay in the medical ICU, on the seventeenth day of admission, her GCS was E4VTM6. Her vitals were stable, and all her counts were within normal limits. She was then shifted to the medical high care unit (MHCU), where IV antibiotics, antipyretics, and multivitamins were continued. She was discharged after 15 days in the MHCU and had normal vitals at the time of discharge. On follow-up one week later, she was doing well.

## Discussion

Scrub typhus is a zoonotic infection that can present with symptoms of fever, headache, myalgia, exanthematous rash, generalized lymphadenopathy, and pneumonia. Gastrointestinal manifestations may include acute abdominal pain, gastric ulcers, hepatitis, jaundice, and hepatosplenomegaly [4]. Apart from these, the presence of an eschar at the site of the chigger bite is pathognomic but its incidence varies from 46% to 85% [5]. Eschar is often absent in individuals from Southeast Asia, which was also the case in our patient [6]. Clinical suspicion and serological tests mark the mainstay of diagnosis of scrub typhus, including the Weil-Felix test, which is highly specific but not very sensitive [7].

Scrub typhus is mostly a self-limiting disease but can lead to fatal illness due to multiorgan involvement if there is a delay in diagnosis and appropriate management. The mortality in such cases can vary from 12% to 50% [8]. Our patient had an APACHE II score of 24, giving her an estimated mortality rate of 30%. Pancreatitis is an infrequent complication of scrub typhus. This was also evident in a previous study that showed that only four out of 136 cases of scrub typhus were complicated by pancreatitis [9]. In our case, pancreatitis was diagnosed due to the presence of typical epigastric pain along with raised pancreatic enzymes. Serum lipase has a higher sensitivity and specificity, up to 82%-100% [10]. However, imaging failed to show significant findings in our case. There are several differential diseases that can cause raised lipase with abdominal pain, which were considered in her case because of her vague clinical picture. She had normal blood sugar and urine ketone was absent so diabetic ketoacidosis was ruled out. Her medical history was negative for inflammatory bowel disease and its symptoms. She had no history of chronic liver disease or peptic ulcer disease symptoms. Acute kidney injury (AKI) could have been the cause of the elevated lipase, but it would not have explained the abdominal pain on its own. The mechanism of pancreatitis in infective conditions is thought to result from bacterial translocation through various proposed mechanisms but is still not completely understood [11].

Liver enzymes are commonly elevated in scrub typhus, but in our case, high prothrombin time (PT)/international normalized ratio (INR) suggested impaired liver function [12]. Similarly, the renal function test showed a high ratio of BUN and creatinine, indicating prerenal azotemia. Both of these findings in association with respiratory fatigue and the need for mechanical ventilation stated the presence of multiple organ dysfunction. She also was in shock, which was unresponsive to fluids. The addition of intravenous antibiotics and vasopressors helped improve her hemodynamic status.

Although the patient belonged to a scrub typhus endemic rural area, the diagnosis of scrub typhus was delayed. Doxycycline was added to her treatment when the diagnosis of scrub typhus was confirmed. Our patient responded well to doxycycline, which may have had some contribution to her improvement. Because of her prolonged period on the ventilator, her disease was complicated with ventilator-associated pneumonia, which was promptly diagnosed and managed. Despite having a poor prognosis, our patient made an uneventful recovery.

## Conclusions

Health professionals in endemic areas should be aware of unusual causes of acute abdomen, including scrub typhus. Scrub typhus can have varied clinical manifestations and serious complications that can be possibly avoided with the timely administration of appropriate antibiotics and supportive measures. In our case, the patients were successfully treated with doxycycline. Pancreatitis in scrub typhus should be studied more extensively to improve our understanding of the disease process and management.
